# The Relationship between Choline Bioavailability from Diet, Intestinal Microbiota Composition, and Its Modulation of Human Diseases

**DOI:** 10.3390/nu12082340

**Published:** 2020-08-05

**Authors:** Natalia Arias, Silvia Arboleya, Joseph Allison, Aleksandra Kaliszewska, Sara G. Higarza, Miguel Gueimonde, Jorge L. Arias

**Affiliations:** 1 Instituto de Neurociencias del Principado de Asturias (INEUROPA), 33003 Oviedo, Asturias, Spain; UO223953@uniovi.es (S.G.H.); jarias@uniovi.es (J.L.A.); 2 Department of Basic and Clinical Neuroscience, Institute of Psychiatry, Psychology and Neuroscience, King’s College London, Denmark Hill, London SE5 8AF, UK; joseph.c.allison@kcl.ac.uk (J.A.); aleksandra.kaliszewska@kcl.ac.uk (A.K.); 3 Department of Microbiology and Biochemistry of Dairy Products, Instituto de Productos Lácteos de Asturias (IPLA-CSIC), 33003 Oviedo, Asturias, Spain; silvia.arboleya@ipla.csic.es (S.A.); mgueimonde@ipla.csic.es (M.G.); 4 Laboratory of Neuroscience, Department of Psychology, University of Oviedo, Plaza Feijóo, s/n, 33003 Oviedo, Asturias, Spain

**Keywords:** choline, TMA, TMAO, non-alcoholic steatohepatitis (NASH), cardiovascular disease (CVD), chronic kidney diseases (CKD), probiotics, gut microbiota, polyphenols, fecal microbiota transplantation

## Abstract

Choline is a water-soluble nutrient essential for human life. Gut microbial metabolism of choline results in the production of trimethylamine (TMA), which, upon absorption by the host is converted into trimethylamine-N-oxide (TMAO) in the liver. A high accumulation of both components is related to cardiovascular disease, inflammatory bowel disease, non-alcoholic fatty liver disease, and chronic kidney disease. However, the relationship between the microbiota production of these components and its impact on these diseases still remains unknown. In this review, we will address which microbes contribute to TMA production in the human gut, the extent to which host factors (e.g., the genotype) and diet affect TMA production, and the colonization of these microbes and the reversal of dysbiosis as a therapy for these diseases.

## 1. Introduction

Choline is an essential nutrient for humans throughout their life. Although humans can produce choline in small quantities through the hepatic phosphatidylethanolamine N-methyltransferase pathway, most individuals need to increase choline ingestion through their diet, in order to prevent deficiency [1,2,3,4]. The main dietary sources of choline include eggs, fish, grains, meat, milk, and their derived products, and to a lesser extent, some vegetables such as soybeans and potatoes [1,3,5,6]. In foods, choline is found as both water-soluble (free choline, phosphocholine, and glycerophosphocholine) and lipid-soluble (phosphatidylcholine and sphingomyelin) forms [7]; however, cooking methods can reduce the amount of choline content in the diet and increase the phosphatidylcholine contribution [8]. Different forms of choline influence the absorption and metabolism of this nutrient throughout development. Indeed, water-soluble forms that are mainly present in human milk enter the portal circulation and reach the liver, while lipid-soluble forms, which can be found in foods, are absorbed and transported through the lymphatic circulation [3,9]. Therefore, the intake of choline varies in accordance with different stages of development.

The majority of people do not meet the dietary requirements for choline, particularly those on vegetarian or vegan diets and pregnant or lactating mothers [1]. Lactation has been associated with an increased choline demand [10], whilst the maternal choline intake could be linked to enhanced placental health [11] and positive neurocognitive effects on the offspring [12]. Therefore, these sub-groups of people should be monitored closely in order to ensure appropriate choline intakes [1,13,14]. 

**Figure 1:**
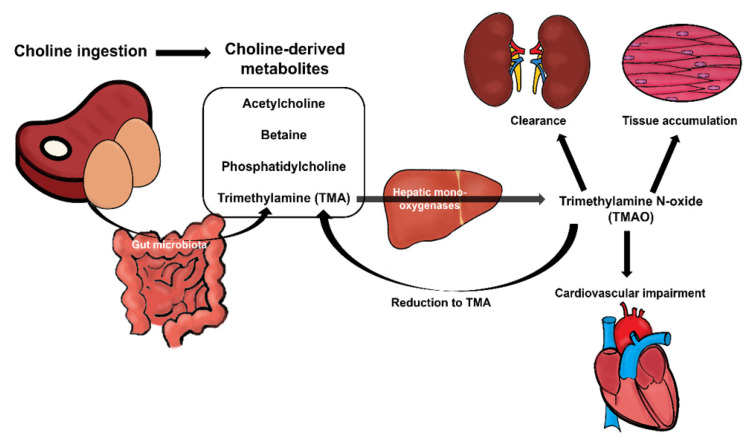
Overview of choline metabolism from the diet. Choline is taken from the diet and gut microbiota trimethylamine (TMA) lyases transform it into TMA. TMA is absorbed by the intestine and delivered to the liver, where TMA is metabolized into trimethylamine N-oxide (TMAO) by host hepatic monooxygenases. Finally, TMAO is distributed to organs, where it can be eliminated (kidneys) and accumulated (tissue). However, it could cause impairment in high concentrations (cardiovascular damage, for a detailed review see Section 5).

Choline metabolism can be divided into four main pathways, which are involved in the synthesis of acetylcholine, betaine, phospholipids, and trimethylamine (TMA; overviewed in Figure 1) [3]. Choline metabolites have a wide range of functions in organisms [1,15]. Choline is catalyzed by choline acyltransferase into acetylcholine, which is key in cholinergic neurotransmission [15,16]. Moreover, choline can be oxidized to obtain betaine, which is an important osmolyte, a methyl donor implicated in the epigenetic regulation of DNA [15,17], and a requirement in the synthesis of phosphatidylcholine [3]. Special attention needs to be given to phosphatidylcholine, the most abundant phospholipid in the body, which is not only a major component of cellular membranes and needed for cell division and growth [18,19], but also plays a role in cell signaling as a donor to synthesize sphingomyelin from ceramide [18,20]. Moreover, sphingomyelin is required for myelination processes in the nervous system [21]. 

Moreover, we want to place a special emphasis on choline metabolism in the large intestine where it is metabolized to TMA by the gut microbiota prior to absorption [22]. The human microbiota harbors trillions of microbes including bacteria, archaea, viruses, phages, yeast, and fungi [23]. It starts to develop in the perinatal period and becomes diverse and complex in adulthood. Several intrinsic and extrinsic factors may affect the microbiota during infancy such as mode of delivery, the gestational age of birth, the infant feeding mode, the maternal diet, environmental factors such as family lifestyle and geographical location and host genetics, and adulthood such as physiological changes of the digestive tract, the modification of dietary patterns, and the impairment of the immune system [24,25]. These factors may disrupt the microbiota composition, causing so-called dysbiosis [26]. The composition of the adult human gut microbiota is mainly represented by the phyla *Bacteroidetes* and *Firmicutes*, followed by *Actinobacteria*, *Proteobacteria,* and *Verrucomicrobia* as minority phyla [25]. The gut is home to more than 1000 microbial species [27] and all of these bacteria encode a microbial gene pool, exceeding the size of the human genome, known as the microbiome [27]. The functions encoded in this microbiome expand the host’s physiological potential, playing an important role in health and disease. This uniqueness of the host microbiota makes it difficult to devise therapies that can work across the population (see Section 6). This has led some authors to consider the intestinal microbiota as a “forgotten organ” [28]. The major function of the gut microbiota is to help in the harvesting of nutrients and energy from our diet. Moreover, it participates in the development of a host’s immune system, brain, and behavior; protects against pathogens; and is a factory of bioactive compounds [29,30]. 

As we have previously pointed out, among these bioactive compounds, the gut microbiota is able to produce TMA, which is absorbed in the intestinal epithelium and subsequently delivered to the liver through the portal circulation. There, it will be metabolized into trimethylamine N-oxide (TMAO) by host hepatic monooxygenases [31,32] such as flavin-containing monooxygenases 3 (FMO3) [33]. Ultimately, TMAO is distributed throughout the body so that it can accumulate in tissues as an osmolyte [34,35] whilst the rest of it is mostly cleared by the kidney, which is in charge of excreting it through the urine (overviewed in Figure 1) [34]. 

Therefore, choline is important for the function and structure of membranes including their signaling, transport, and repair [36,37,38]. Moreover, as we have previously mentioned, it is the key in the synthesis of acetylcholine, methylation, gene expression [39], and liver and muscle function [36]. However, it has recently gained attention due to its association with adverse health outcomes [3]. Currently, the identification of the type of bacteria involved in TMA synthesis and the mechanisms by which choline and its metabolites TMA and TMAO contribute to the risk of disease need to be properly evaluated, in order to understand choline’s impact on health.

In this review, we will revisit the relationship between gut microbiota populations and circulating TMA and TMAO levels, highlighting not only the microbiota genetics behind these changes but also how host genetics can influence the gut microbiome. We will also discuss the relationship between choline-microbiota changes and their impact on different diseases as well as explore microbial modulation as a potential therapeutic treatment.

## 2. The Impact of Gut Microbiota on Choline Metabolism

**Figure 2:**
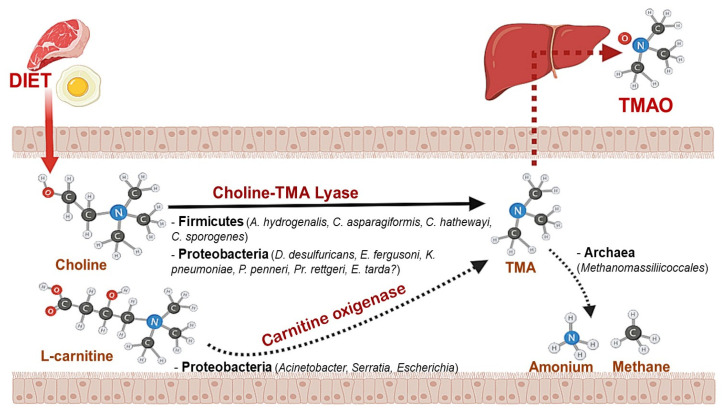
Microorganisms involved in the metabolism of dietary choline and other trimethylamine-containing compounds. Following ingestion of foods containing choline/lecithin, or L- carnitine, certain intestinal microorganisms metabolize these compounds to trimethylamine (TMA) by different metabolic pathways. TMA can then be absorbed and transformed into trimethylamine N-oxide (TMAO) in the liver, or it can be reduced by methanogenic archaea in the gut to produce methane and ammonium.

The ingestion of food containing choline or other trimethylamine-containing compounds is followed by the synthesis of TMA in the gut by microorganisms including both Gram-positive and Gram-negative bacteria (Figure 2). Therefore, the magnitude of the production of TMA is influenced by the composition of the microbiota of the individual. It is important to note that only a minor fraction of the microorganisms present in the intestine (less than 1%) harbor the genes required for TMA production [40]. However, even very low concentrations of these microorganisms seem to be sufficient for TMA production, which illustrates the importance of the gut microbiota in this context [41]. Indeed, the presence of increased TMA and TMAO levels has been associated with higher activity of bacterial members of the phylum *Firmicutes* and *Proteobacteria*, which are known producers of this metabolite [40,41]. Moreover, TMA and TMAO levels have been linked to an elevated *Firmicutes*/*Bacteroidetes* ratio with higher levels of *Firmicutes* and lower levels of *Bacteroidetes* [42,43] due to the inability of *Bacteroidetes* to produce TMA [44,45].

Recently, the genetics that underpin TMA production in the microbiota has received a great deal of attention. This has led to the identification of different gut microbial genes and gene-clusters involved in the catabolic reactions converting dietary compounds into TMA. Among them, the cluster *CutC*/*CutD*, which codes for the choline TMA-lyase and its activating protein [46], seems to be key in the intestinal environment [47], whereas the carnitine oxygenase (*CntA*) contribution to TMA production is lower [48]. 

Moreover, betaine (choline-derived metabolite) reduction by the action of the microbial enzyme glycine betaine reductase coded by the gene *GrdH* seems to play a minor role in TMA production in the gut [40]. This also seems to be the case for the production of TMA via the intermediate γ-butyrobetaine derived from carnitine by the action of the *YeaW*/*YeaX* cluster [49]. 

Although the *CutC*/*CutD* cluster appears to be the most widely distributed mechanism for TMA production from choline, it has been demonstrated that a quarter of TMA production is linked to *CntA*, which is present in lower amounts [32]. Moreover, a recent study [40], in which mammal species were compared, revealed that herbivores harbor a lower relative abundance of *CutC* while lacking *CntA*. These results point to the influence of the evolving relationship between host genetics and diet in the prevalence of these genes, as illustrated by the differences between herbivores and carnivores. It is also important to note that our knowledge in this area is limited and some pathways involved in TMA production are still unknown. Supporting this, Wu et al. [50] found no association between *CntA* and serum TMAO levels after carnitine consumption, suggesting that other pathways such as the γ-butyrobetaine pathway may also be of relevance in the gut environment. In addition, the lack of any known TMA-producing gene in *Edwardsiella tarda*—a known TMA-producing microorganism—also suggests the existence of other unknown pathways [47].

Regarding the microbial taxa harboring these genes, it was found that the cluster *CutC*/*CutD* is the most widely distributed, not only being present in a variety of intestinal microorganisms, in particular in *Firmicutes*, especially the *Clostridium* cluster XIVa and *Eubacterium* strains, but even in some *Actinobacteria* and *Proteobacteria* [40]. *GrdH* also seems to be mainly distributed among *Firmicutes*, but it has been found in some *Spirochaetes*. On the contrary, the presence of *CntA* and *YeaX/Y* is mainly associated with the bacterial phyla of *Proteobacteria*, with *Escherichia* and *Acinetobacter* being the main genera harboring these genes in the human gut [40]. 

**Figure 3:**
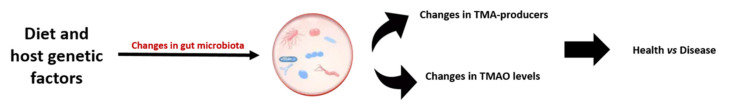
Overview of factors affecting gut microbiota and direct effects on TMAO levels.

In the specific case of choline metabolism, TMA is formed through the action of the microbial choline TMA-lyase (Figure 3) [51]. Specifically, the intestinal bacteria in charge of TMA production from choline include *Anaerococcus hydrogenalis*, *Clostridium asparagiformis*, *Clostridium hathewayi*, *Clostridium sporogenes*, *Desulfovibrio desulfuricans*, *Escherichia fergusoni*, *Ed. tarda*, *Klebsiella pneumoniae*, *Proteus penneri*, and *Providencia rettgeri* [41,52].

Once TMA is formed, it will be subsequently oxidized into TMAO in the liver through the enzyme FMO3 [53]. It is worth noting that some bacteria from the phylum *Proteobacteria* may also be able to metabolize the TMAO ingested via the diet into TMA via TMAO reductase using metabolic retroconversion [54]. Finally, it has recently been found that intestinal archaea such as some members of the order *Methanomassiliicoccales* are able to reduce TMAO to methane [55]. Therefore, a current area of research relies on the use of such microorganisms as potential probiotics, in order to reduce the circulating levels of TMAO, which have been associated with an increased cardiovascular disease risk.

## 3. Diet Impact on Microbiota-Choline Metabolism

It has been demonstrated that the composition and diversity of gut microbiota can be influenced by the diet, since it is, in turn [56], an important source of variability in serum TMAO levels. In this regard, long-term individual dietary habits have been proven to influence microbiota enterotypes [57,58]. Indeed, Wu et al. [58] observed that a Western diet consumption—typically represented by a high consumption of animal proteins, saturated fats, and low fiber—was associated with the *Bacteroides* enterotype, whereas a carbohydrate-based diet mainly consumed by agrarian societies, was linked to a *Prevotella* enterotype. Western diets are characterized by animal products such as liver, pork meat, and eggs, which contain large amounts of choline [59] and are known not only to increase blood and urine TMAO levels [41] but even have an effect on the gut microbiota. In fact, Manor and colleagues [60] observed a positive correlation between intestinal microbial clades such as *Neisseriaceae* or *Desulfovibrio* and TMAO levels, which were also elevated in symptomatic coronary vascular disease (CVD) patients and those consuming an animal-based diet. Another study from Cho et al. [42] reported that men with elevated levels of TMAO in the body after consuming dietary eggs tended to have a higher abundance of *Firmicutes*; meanwhile, individuals with lower levels of TMAO exhibited a higher abundance of *Bacteroidetes*. 

Similar differences in microbiota and TMAO levels have been reported between vegetarian and omnivorous diets, which were also accompanied by a lesser ability to produce TMA in vegetarians [61]. These observations reinforce the potential of modulating dietary habits to reduce the risk associated with high TMAO levels. 

Nevertheless, due to the importance of choline as an essential nutrient, choline-deficient diets may also induce gut microbiota alterations and health problems [62,63,64,65]. Indeed, a human trial in which the choline intake was controlled demonstrated that the gut microbiota composition changed with dietary choline levels and specific alterations in *Gammaproteobacteria*, and *Erysipelotrichi* members were associated with changes in liver fat during choline depletion [66]. These results confirm the impact of diet on both gut microbiota and TMAO levels [61,66]. In addition, it has been reported that FMO3 expression is closely related to the composition of intestinal flora [53,67]. The changes observed after choline-dietary interventions, together with the putative host´s genes, could be used for predicting and modulating the risk of developing diseases related to this nutrient (overviewed in Figure 3). 

## 4. Host Genotype Impact on Microbiota Choline Metabolism

The concentration of TMA/TMAO in plasma has been linked to the composition of intestinal microbiota and differences among microbiota enterotypes [57] have been proposed [68]. Nevertheless, it may be considered that the high inter-individual variability in the composition of the intestinal microbiota results in a large variability in its enzymatic capabilities [69]. Furthermore, this inter-individual variability is linked to different factors including the host’s genetics.

Over a period of decades, different studies have tried to understand how a host’s genetic background may influence the overall microbiome. The proportion of phenotypic variation in a trait that is attributable to genetic variation among individuals is known as heritability [70]. Among the gut microbiota, a few bacteria and archaea have arisen as heritable and have been associated with host genes. In line with this, twin studies have observed a higher similarity in the gut microbiota composition between monozygotic than dizygotic twins, which is attributable to shared genes. These studies also identified heritable bacteria including *Christensenellaceae* (later associated with a low body mass index), the methanogenic Archaea *Methanobrevibacter smithii*, and the genus *Blautia* [71,72]. Moreover, microbiome genome-wide association studies have also identified associations between human genes and the gut microbiome such as the lactase gene *LCT* and the abundance of *Bifidobacterium* [73,74] or the vitamin D receptor gene and microbial diversity [75].

Focusing on how host genetics can influence the gut microbiome in a disease context, several associations between specific single nucleotide polymorphisms or genetic variants of host genes and intestinal bacteria have been identified (Table 1). These support the idea that host genes involved in the digestion of sugars, dietary preference, and immunity can alter the composition of the gut microbiome [76,77,78,79,80,81]. There is a lack of knowledge on the relationship between host genetics and gut microbiota in choline-mediated diseases. However, regarding liver diseases, one study has identified a quantitative traci loci (QTL) in chromosome 7 of the mouse genome (MM7) that showed genome-wide linkage with the relative abundance of *Turicibacter*, which was overlapped with the HCS1 QTL for susceptibility to murine hepatocellular carcinomas [82]. This opens up the door to investigate how specific host genes mediate an altered gut microbiota composition, contributing, in this way, to its abnormal function and the development of diseases such as those studied in this review. 

Moreover, genotype-associated differences in TMAO and TMA levels were also suggested in a murine model. Romano and colleagues [41] observed higher levels of serum TMAO in female mice, which were associated with higher relative abundances of TMA-gut microbial producers rather than their male counterparts. These gender-associated variances were attributed to the higher hepatic flavin-containing mono-oxygenase (FMO) activity in females, in line with other studies [53,69]. However, contradictory results were found for the TMAO concentration related to gender differences in humans, which have been linked to several confounding variables such as age, body mass, and blood pressure [34]. 

## 5. Choline Intake and Its Relationship to Disease

**Figure 4:**
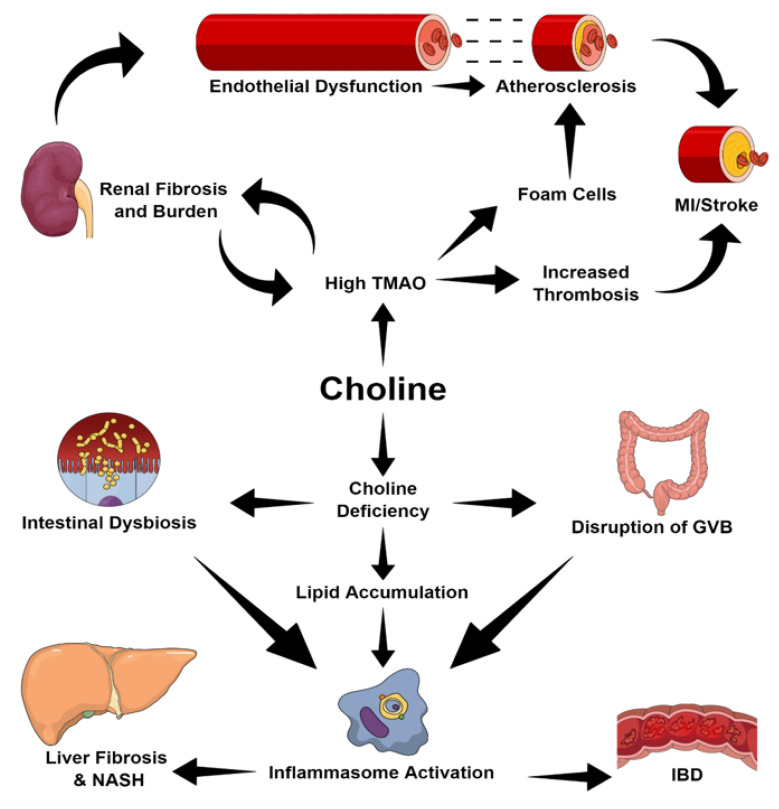
An overview of the ways in which choline intake may cause disease. Abbreviations: GVB, gut vascular barrier; IBD, inflammatory bowel disease; MI, myocardial infarction; NASH, non-alcoholic steatohepatitis; TMAO, trimethylamine-N-oxide.

As previously mentioned, TMAO is correlated with disease and all-cause mortality [90,91]. Low serum TMAO induced by choline-deficits diets is associated with non-alcoholic steatohepatitis (NASH), while high concentrations of TMAO are associated with CVD and chronic kidney disease (CKD). Here, we will discuss the mechanisms through which varying levels of choline intake cause disease (overviewed in Figure 4).

Average plasma TMAO in humans is around 3.3 µM; however, this can vary greatly after the intake of certain foods that are rich in choline and returns to the baseline within a few hours [35]. CKD sufferers often have persistent exaggerated levels of plasma TMAO, although, following a kidney transplant, the TMAO concentration was shown to reduce to almost control levels [91,92]. However, it remains unclear whether this is simply a marker of renal impairment or whether TMAO plays an active role in renal function. Tang et al. [93] found an upregulation of phosphorylated Smad3, a profibrotic marker, and KIM-1 (kidney injury marker) in male mice fed choline- and TMAO-supplemented diets [94]. This was accompanied by increased collagen deposition and tubulointerstitial fibrosis, suggesting that high levels of TMAO may result in renal pathology and subsequent impairment such as endothelial dysfunction [93,95]. 

The inability to regulate the vascular tone is an early marker of CVD and may lead to atheroma-development. Mice fed a choline-supplemented diet displayed significantly less reverse cholesterol transport with reductions in mRNA of Cyp7a1 and Cyp27a1. This attenuated the conversion of cholesterol into bile acids, resulting in cholesterol build-up [68]. Furthermore, increased TMAO levels upregulate macrophage scavenger receptors such as CD36 and the recruitment of leukocytes [34,96,97]. Together, these promote the formation of foam cells, which build-up to form atherosclerotic plaques [98]. With the build-up of atheroma, myocardial infarction and stroke present the greatest risk, with thrombi more likely to occlude the entire arterial lumen [31,90,99]. Following arterial injury, choline-rich diets reduce the time required for vessel occlusion, which is accomplished by the TMAO-induced release of intracellular calcium ions into the cytosol of platelets, thereby activating platelets and promoting adhesion [31,99]. 

Rises in TMAO related with CKD and CVD have been associated with an increased abundance of TMA-producing bacteria [60]. Romano and colleagues [100] have suggested that choline-consuming microbiota compete with the host for choline, in order to produce TMA, and thereby reduce the bioavailability of this nutrient. Subsequently, this leads to a reduction in S-adenosylmethionine (SAM) methyl-donor groups, for which choline is a major source. The loss of methyl donors may cause epigenetic dysregulation and hypomethylation of the host’s genome [100,101]. Indeed, the global hypomethylation of DNA has been associated with CKD and atherosclerosis [102,103]. Equally, hypermethylation has also been correlated with CKD and therefore illustrates the complexity of epigenetic regulation and the need for further research [104,105]. Interestingly, Romano [100] found that maternal epigenetic profiles can be passed on to offspring through pregnancy and may alter the offspring’s epigenome in utero. This may have long-term post-natal effects such as behavioral and cognitive changes [100,101,106,107]. The potential for nutritional intake to have inter-generational impacts through epigenetic profiles is highly undocumented territory and may present one way through which parent biology may affect the offspring long-term, independent of the genotype.

Choline-deficient diets have also been associated with NASH and may result in obesity and hyperglycemia [64,65,66,108]. Research indicates the role of choline-deficient diets in intestinal dysbiosis by reducing the microbiota diversity and altering the microbial population representation within the microbiome [64,65,109]. Subsequent transplantation of the microbiome of NASH individuals is sufficient in inducing NASH in otherwise healthy individuals [65,110]. 

Choline-deficient diets interfere with and disrupt the intestinal barrier [64,111,112]. Downregulation of the Wnt/β-Catenin pathway following a methionine-choline-deficient diet (MCD) results in disruption of the gut vascular-barrier (GVB) and upregulation of PV1, a marker of GVB [112,113]. In addition, zona occludens-1, a tight junction protein and permeability marker, is lower in those receiving a MCD diet [109,114]. This increases bacterial infiltration from the gut into peripheral organs such as the liver and leads to steatosis and lipid accumulation. 

Lipid accumulation may be a result of reductions in fatty acid esterification genes, which prolong the half-lives of injury-inducing lipids [115,116]. Moreover, the downregulation of genes in very-low-density lipoprotein (VLDL) secretion and reduced phosphatidyl choline and triglyceride contents of VLDLs promote the build-up of cholesterol and fats in the liver [116,117,118]. These surplus fatty acids may promote the activation of inflammasomes [119].

Dysregulation in the microbiome is correlated with the activation of the Nod-like receptor protein 3 (NLRP3) inflammasome which is necessary for fibrosis in developing NASH pathology [120]. Moreover, NLRP3 activation may be involved in patients suffering from inflammatory bowel disease (IBD). In this regard, it has been demonstrated that an elevation of NLRP3 levels exacerbates inflammation in colitis mouse models for IBD [121]. Despite this, NLRP3 deficiency has been seen to both exacerbate and attenuate intestinal inflammation, highlighting the complexity of NLRP3’s role in inflammation [122].

Abnormal choline-intake is involved in the pathogenesis of major debilitating diseases such as CKD, CVD, and NASH (Table 2). Despite an overwhelming amount of evidence demonstrating correlations between choline, and its metabolite TMAO, in terms of disease onset, it is not uncommon to see conflicting data and reports of no correlation between them. Some inconsistencies revolve around L-carnitine and betaine showing protective effects against CVD and therefore casts doubt on TMAO’s involvement. However, we cannot rule out the possibility that betaine or L-carnitine have some regulatory effect on TMAO that reduces the proatherogenic effect of TMAO just as much, as these studies suggest that TMAO is beneficial [123,124]. As a result, these present an inherent problem when trying to draw conclusions about the effects of choline intake on disease. Historically, it has been impractical to measure choline levels alongside TMAO and TMA concurrently. Despite TMA being the main intermediate in the formation of TMAO, it is not routinely tested for (with TMAO being preferred). It is possible that studies that report no correlation with TMAO may instead show correlations between TMA and disease [125,126]. In line with this, a new methodology by Wu and colleagues [127] allows for the simultaneous measurement of TMA and TMAO and provides the opportunity to establish the exact role and associations of these choline metabolites and disease in future research.

## 6. Potential Therapies in Choline-Related Diseases

**Figure 5:**
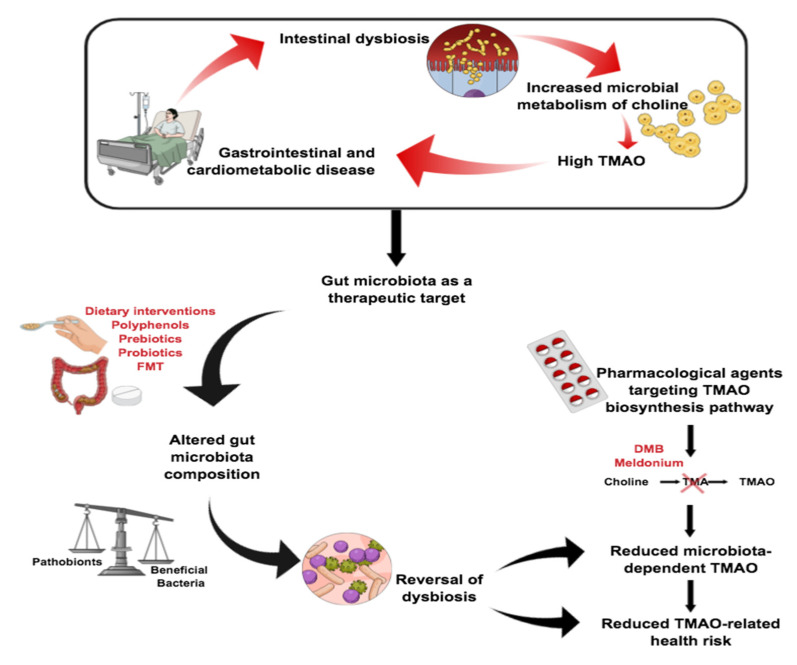
Overview of microbiota-targeting therapies for the treatment of gastrointestinal and cardiometabolic disorders Abbreviations: FMT, fecal microbiota treatment; DMB, 3,3-Dimethyl-1-Butanol.

Data from associative and mechanistic studies draw a strong link between dysbiosis and pathobiotic bacteria in the gut, the microbiota-dependent production of TMAO, and an increased risk of cardiometabolic and gastrointestinal disorders [98,113,150,151,152,153]. Since many of those disorders lack effective treatments, the gut microbiota has emerged as an attractive therapeutic target (see Table 3). Interventions designed to reverse dysbiosis and lower TMAO levels by (i) inducing alterations in the microbiota composition, which boosts the abundance of beneficial bacteria and reduces the TMAO production capacity, and (ii) directly disrupting the TMAO biosynthesis pathway are being developed as potential treatments (overviewed in Figure 5).

As we have previously described, diet is an important lifestyle determinant of gut health. This makes dietary modification a potentially easy and relatively risk-free strategy for reducing TMAO-related health risks. Studies investigating the effect of diet on intestinal microbiota and TMAO levels have largely focused on the Mediterranean diet and veganism, both of which are rich in plant-derived polyphenols, high in fiber, and associated with multifaceted health benefits [154,155]. Epidemiological studies have reported an inverse correlation between habitual adherence to the Mediterranean diet and urinary [156] as well as plasma [157] TMAO levels in the Southern European population. However, six months of Mediterranean dietary intervention failed to reduce the fasting TMAO concentration in healthy North American volunteers at risk of colon cancer [158]. This discrepancy may be explained by population differences, which are known to affect the microbiota composition [159]. It also suggests that short-term interventions may be insufficient to induce re-modeling of intestinal microbiota shaped by years of the habitual consumption of a Western diet, known to cause dysbiosis and elevate TMAO levels in rodents [130].

Similarly, long-term adherence to a vegan diet is linked with a diminished capacity to biosynthesize TMAO from the dietary TMAO precursor L-carnitine [68], but studies comparing the baseline plasma concentration of TMAO between vegans and omnivores have yielded conflicting results [68,160]. This discrepancy may be explained by differences in inclusion criteria, with studies applying stricter criteria taking into account individuals’ dietary history reporting more significant changes, suggesting an inverse correlation between years’ spent vegan and TMAO levels. Notably, background diet was found to influence the meat consumption-induced TMAO concentration in a porcine model, with pigs fed high-fiber diets showing a significantly lower increase in TMAO levels compared to those kept on a high-fat Western-like background diet [161]. This emphasizes the modulatory effect of a habitual diet on short-term dietary intervention-related health outcomes.

‘Functional foods’ can exert beneficial effects on human health beyond their basic nutritional value. One example is given by prebiotics, defined as non-digestible food components selectively degraded by bacteria in the gut [162] that act to promote the growth of health-benefiting microbes. Non-digestible carbohydrates are the most commonly prescribed prebiotics, with a plethora of clinical data supporting their role in promoting microbial diversity [163]. Notably, prebiotic supplementation with soluble dietary fiber not only boosted the abundance of beneficial bacteria, but also significantly diminished TMA and TMAO metabolism (by 40.6% and 62.6%, respectively) in mice fed a red meat rich diet. Moreover, this was accompanied by a marked reduction in weight, decrease in energy metabolism, and improved lipid and cholesterol markers [164]. Similarly, in humans, the consumption of an arabinoxylan-oligosaccharide-enriched prebiotic extract as part as an intervention trial in overweight adults was shown to increase the prevalence of *Prevotella* normally associated with non-Western diets low in processed foods [165]. Notably, the *Prevotella* abundance was also associated with an increase in short chain fatty acids and a rise in plasma phosphatidylcholine, indicative of a reduced availability of choline for TMA biosynthesis and a potential protective role in promoting metabolic health [165]. Likewise, long-term adherence to a diet enriched with dietary fiber was associated with a reduction in the TMAO concentration, accompanied by changes in the gut microbiota and improved metabolic health in obese children [166].

Polyphenols are phytochemicals produced as secondary metabolites in plants and constitute another class of chemical compounds of dietary origin with extra health benefits, known for their potent gut microbiota modulating properties. Notably, dietary supplementation with resveratrol (a stilbenoid polyphenol) increased the abundance of *Lactobacillus*, reduced the levels of TMAO and attenuated the atherosclerosis phenotype of ApoE^-/-^ mice fed a high-choline diet [167]. Flavonoids present in oolong tea extracts and citrus peel were reported to have similar *Lactobacillus*-boosting effects, accompanied by a reduction in the carnitine-induced increase in TMAO plasma levels in mice [168]. A recent double-blind randomized trial evaluating the TMAO-reducing efficacy of Taurisolo, a polyphenolic-rich pomace extract, found that it induced a significant reduction in TMAO relative to the placebo (63.6% vs. 0.54%) four weeks post-intervention [169]. However, additional mechanistic and human intervention studies are needed to further elucidate the relationship between polyphenols, microbiota-dependent TMAO levels, and human disease.

Another strategy for targeting TMAO is the use of probiotics (i.e., the ingestion of living microbes in adequate amounts that exert beneficial effects on human health) [170], with studies reporting promising microbiota re-modeling and TMAO-reducing effects in animal models. Eight weeks of dietary supplementation with *Lactobacillus paracasei* F19 protected rats from oxidative stress-induced liver damage by restoring the intestinal barrier and microbiota diversity [171], while gut colonization with *M. smithii* bacterial species was associated with a reduction in the TMAO concentration and attenuation of atherosclerosis in ApoE^-/-^ mice [172]. *Enterobacter aerogenes* is another bacterial strain that has been shown to effectively reduce plasma TMAO and cecal TMAO levels by shifting the ratio of commensals and pathobionts in mice on a high-choline diet [173]. While the results are promising, it is important to apply caution when extrapolating findings from animal models into a clinical setting as the microbiome of rodents and humans are very different [174] and inter-personal differences in the microbiota composition resulting from the habitual diet and genetic make-up need to be considered, as previously explained.

A more recent approach to reversing dysbiosis is fecal microbiota transplantation (FMT), which is a type of fecal bacteriotherapy that involves complete replacement of the recipient’s indigenous microbiome with healthy microbiota from a carefully selected donor. Despite positive results in pre-clinical studies, the method has yielded limited clinical success with antibiotic-resistant *Clostridioides difficile* infection being the only human condition for which FMT is currently approved [175]. However, it is currently unclear whether gut colonization is sufficient for clinical success in diseases beyond *C. difficile* infection. In this regard, a small FMT clinical study has shown to reverse gut dysbiosis but failed to induce remission in patients with chronic active ulcerative colitis [176]. Similarly, FMT in a double-blind randomized pilot study of 20 obese patients with metabolic syndrome elicited remodeling of the gut microbiota composition toward that of the vegan donor in some but not all recipients [160]. However, even in individuals that responded to the treatment, changes in the microbiota did not translate into a reduction in the TMAO production capacity nor alleviation of the atherosclerotic burden. In the future, precision-medicine-derived approaches could be adapted to transform FTM from an untargeted global microbiome replacement therapy into a more refined individual- and disease-specific intervention ensuring host–recipient compatibility by the application of standardized screening protocols and reliable donor selection criteria. However, this is contingent on developing an improved understanding of the causative relationship between individual bacterial strains and disease susceptibility.

Pharmacological agents designed to disrupt microbial metabolite biosynthesis cascades include a structural analogue of choline 3,3-Dimethyl-1-Butanol (DMB) and the anti-ischemic drug meldonium. DMB works to reduce TMAO levels and attenuate choline diet-induced atherosclerosis by inducing the non-lethal inhibition of TMA lyase in the gut, which is hypothesized to be associated with a reduced risk of resistance compared to antibiotic treatment, which causes considerably more selective pressure [177]. Despite its success in a pre-clinical model, the drug has never been tested in humans due to potential off-target adverse effects. Meldonium is a safer alternative, but clinical data suggest that it only inhibits TMAO produced from carnitine, and not choline [178].

Together, dietary interventions, the use of prebiotics/ probiotics, FMT, and pharmacotherapy represent an exciting potential avenue for the treatment of human diseases associated with dysbiosis and elevated TMAO levels. However, the success of microbiome-targeting therapies is contingent on developing an improved mechanistic understanding of interactions between the gut microbiota and disease pathogenesis. There is clearly a need for better designed randomized double-blind human trials with longer follow-up periods and bigger sample sizes. On the other hand, promoting lifestyle modifications that improve gut health is of significance as evidence suggests that long-term dietary habits and the state of the microbiome pre-treatment are important determinants of clinical success.

## 7. Conclusions and Future Directions

TMAO is a metabolite of dietary choline and is therefore dependent upon the consumption of precursors, host genetics, and gut microbial enzymatic activity. In this review, we described specific microbial enzymes involved in TMA production pathways as well as the microbes carrying the genes for these enzymes [135]. It is worth noting that the production of TMA/TMAO relies not only on gut microbiota, but also on host genetics, co-metabolism, and diet consumption. This gives rise to large inter-individual variability, raising the possibility that geographic differences in dietary patterns or gut microbial composition may affect the generalizability of results or require modifications to dietary intake [193].

However, important questions remain. Archaea has not received the same level of attention as bacteria, despite potentially being important in nutrient metabolism including TMA production, as supported by Fu et al. [194]. Regarding studies supporting the associations between TMAO and disease such as CVD, it is important to note that they have been mainly performed in people already with a disease, or at high risk of CVD, renal, or metabolic outcomes [195]. Therefore, it remains unknown as to whether this relationship is present in the general population. Moreover, TMAO levels rely on the consumption of dietary precursors, which is an association that should be further studied [193].

Another emerging field is the role of choline as an epigenetic modifier of the genome by changing the availability of methyl-donors such as S-adenosylmethionine (SAM). SAM synthesis depends on dietary choline ingestion and modulates neuronal gene expression and brain function. Therefore, the identification and implementation of effective nutritional strategies early in life could improve cognition and mental health [196].

Therefore, the study of the gut microbiome may significantly enhance our understanding of nutrient metabolism and specific pathways by which diet can influence health and cognition. This may facilitate the adoption of a individualized nutrition-based approach to target gut microbial structure and function as well as the potential to alter different metabolites such as TMAO production.

## Figures and Tables

**Table 1:** Host genetic variants with an effect on the gut microbiota (adapted from Spor et al., 2011 [70]).

Host Genetic Variant	Gut Microbiota Impact	Diseases or Adverse Phenotypes
***MEFV*** encodes pyrin, one of the regulators of innate immunity [83]	Changes in bacterial community structure, mainly in *Bacteroidetes*, *Firmicutes*, and *Proteobacteria*; loss of bacterial load and diversity depended on the allele carrier status of the host [83]	Mutations in *MEFV*: Familial Mediterranean fever (autoinflammatory disorder) [83]
**APOA1** major component of the high-density lipoprotein (HDL) [84]	Changes in community structure in APOA1-deficient mice [84]	SNPs in APOA1: risk of obesity, cardiovascular disease, and hyperlipidemia [84]
**MyD88** adaptor for multiple innate immune receptors that recognize microbial stimuli [85]	Change in distal gut microbiota composition: higher *Lactobacillaceae, Rikenellaceae,* and *Porphyromonadaceae* abundances in MYD88-deficient mice [85]	Loss of MYD88: comprised innate immune response to pathogens [85]
**NOD2** intracellular pattern recognition receptor of muramyl dipeptide constitutively expressed in human Paneth cells [86]	Increased load of commensal resident bacteria in Nod2-deficient mice and shifts in the relative frequencies of *Faecalibacterium* and *Escherichia* [86]	Mutations in NOD2: risk factor for Crohn´s disease and diminished ability to prevent intestinal colonization of pathogenic bacteria [86]
**HLA** proteins that are encoded by the major histocompatibility complex (MHC) gene complex in humans [87]	Correlation between higher genetic risk and bacterial groups: *Streptococcus-Lactococcus, E. rectale-C. coccoides, Clostridium, Bacteroides-Prevotella* groups and total Gram-negative bacteria [87]	Variation in HLA genes: risk of celiac disease [87]
***SLC39A8*** encodes alanine or threonine at position 391 in the zinc transporter solute carrier family 39, member 8 protein [76]	Association between the risk locus that carries *SLC39A8* and the abundance of *Anaerostipes, Coprococcus*, and *Lachnospira* [76]	Variants of *SLC39A8*: associated with inflammatory bowel disease (IBD) and distinct phenotypes including obesity, lipid levels, blood pressure, and schizophrenia [76]
*α-**defensin*** [88]	Alpha-defensin-dependent changes in microbiota composition, but not in total bacterial numbers. Lower segmented filamentous bacteria numbers [76].	Changes in the copy numbers in defensin genes: Crohn’s disease [88].
**IgA locus**> [89]	Predominant and persistent expansion of segmented filamentous bacteria throughout the small intestine in activation-induced cytidine deaminase, which produces an absence of IgA [89].	Lack of IgA: higher incidence of inflammatory bowel diseases [89]

**Table 2:** Summary of the association between disease, TMAO levels, and their effects on metabolites and microbiota.

Disease and Its Associated Constituents	TMAO Levels	Effect on Metabolites	Effect on Microbiota/Additional Comments
**CKD**	↑	↑ Phosphorylated Smad3, Cystatin C, Kim-1 [93,128], Nox-4, TNF-α, IL-1β [128].	↑ TMA-producing bacteria [60,129]: *Desulfovibrio* [60,130], *Dehalobacterium* [60,106], *Clostridiaceae* [60,68], *Christensenellaceae* [60,131], *Proteobacteria* [132].
**Endothelial Dysfunction** **(Seen in CKD and CVD models)**	↑	↑ IL-6, TNF-α, hsCRP, HMGB1 [91,96,97,133,134,135]	↓ Firmicutes, *Actinobacteria, Roseburia, Coprococcus, Ruminococcaceae, Prevotella* [132].
		↑ VCAM1 [97,136]	Increases in TMA-producing bacteria are associated with high-TMAO levels and therefore, also present in CVD with similar levels of TMAO
		↓ eNos [133]	.
		↓ IL-10 [134]	
		↑ Superoxide [133]	
		↑ NLRP3, Caspase-1, IL-1β [137]	
**Atherosclerosis**	↑	↓ Cyp7a1; Cyp27a1 [68] ↑ CD36 [34,96,97,98] ↑ Leukocyte Recruitment [34,97,98] ↑ Galectins [60] ↑ TNF-α, HMGB1 [138] ↑ IL-1β [139]	↑ *Prevotella*, *Tenericutes* [68], *Allobaculum* [99].
			↓ *Lachnospiraceae, Candidatus Arthromitus, Peptococcaceae* [99].
			Changes in *Clostridiales* [139].
		
		
		
**Vessel Occlusion**	↑	↑ Platelet (Ca^2+^)_i_ [31,99]	*Prevotella*/*Cyanobacteria* [99] negatively correlates with vessel occlusion time. *Peptococcaceae* positively correlates with vessel occlusion time.
		↑ TF, Thrombin [138]	
		↑ CD36 [140]	
**MCD-Induced NASH**	↓	↑ PV1 [112,113] ↓ ZO-1 [64,109,111,114] ↓ Phosphatidyl choline/Triglyceride in LDLs [116,117,118] ↑ Caspase-2 [116] ↑ TGFβ, αSMA, COL1A1, CRP2 [141,142] ↑ NLRP3, Caspase-1 [116,118,121,141,143] ↑ IL-18, IL-1β [119,141,142,144,145,146]	↓ *Verrucomicrobia, Actinobacteria, Proteobacteria, Bifidobacteriaceae* [64], *Lactobacilli* [65,110], *Akkermansia* [65].
			↑ *Lachnospiraceae, Barnesiella, Allobaculum* [110,118], *Ruminococus* [65,147], *Bacteriodetes* [64,147], *Tenericutes*, *Desulfovibrio, Enterobacteriaceae* [64], *Firmicutes*, *Helicobacteraceae* [64,118].
			*Allobaculum* negatively correlated with ZO-1 [118].
		
		
		
		
**IBD**	↓	NLRP3 changes as detailed under NASH above ↓ ATG16L1, LC3-II, P62 [148]	↑ *Firmicutes, Proteobacteria, Verrucomicrobia, Fusobacteria* [149]
			↓ *Bacteriodetes, Cyanobacteria* [149].

Abbreviations: CKD, chronic kidney disease; CVD, coronary vascular disease; TNF-α, Tumor Necrosis Factor α; IL- (1β, 6, 10), Interleukin-; hsCRP, high sensitivity C Reactive Protein; HMGB1, High Mobility Group Box 1; VCAM1, Vascular Cell Adhesion Protein-1; eNOS, endothelial Nitric Oxide Synthase; NLRP3, Nod-like Receptor Protein-3; (Ca2+)I, Intracellular Calcium Ions; TF, Tissue Factor.

**Table 3:** Dysbiosis, microbiota-dependent TMA production, and current treatments in choline-related diseases.

Disorder.	Dysbiosis	TMA Production	Other Components	Therapy	Effects
CVD/atherosclerosis	Decreased microbial diversity; reduced abundance of bacteria from *Lachnospiraceae* family; correlation between abundance of *Candida, Campylobacter, Shigella,* and *Yersinia* pathobions and heart failure severity [179,180]	Increased [98]	Increased plasma and urine levels of TMAO; increased expression of FMAO3; dietary choline-induced formation of foam cells in mice models [98,181]	Resveratrol	Microbiota re-modeling; reduction in TMAO levels [167].
				SCFAs	Vasodilation; decreased plasma TMA levels and TMA:TMAO ratio; increased microbial diversity [182].
				DMB	Reduction of TMAO and amelioration of atherosclerotic burden in ApoE-/- mice; suppression of TMA production in-vitro [177].
				Probiotic supplementation with bacteria from *M. smithii*/*E. aerogenes* strains	Reduced plasma/cecal levels of TMAO and amelioration of atherosclerosis in ApoE-/- mice; increased abundance of beneficial bacteria [172,173].
				Allicin	Reduction in carnitine-induced elevation of plasma TMAO levels in mice, microbiota re-modeling [183].
				Antibiotic therapy	Plasma TMAO levels were greatly reduced during antibiotic therapy and quickly recovered after the treatment was stopped [173].
Inflammatory bowel disease	Broad gut microbiota dysbiosis; reduced microbial diversity; decreased abundance of *Firmicutes* and *Bacteroides;* increased abundance of *Gammaproteobacteria* [86,184,185]	Increased [186]	Decreased levels of serum choline; reduced TMAO plasma levels in IBD patients vs. control population [176,186]	FMT	Re-establishment of healthy gut microbiota but failure to achieve disease remission in chronic colitis patients [176].
NAFLD	Increased abundance of *Erysipelotrichaceae,* reduced abundance of *Gammaproteobacteria;* reduced cecal abundance of lactic acid bacteria *Bifidobacterium* and Lactobacillus [66,187]	Increased [188]	Low choline bioavailability [66]	L-carnitine supplementation	Decreased lipid accumulation and oxidative stress injury, attenuation of systemic inflammation and inhibition of fibrosis progression in mice fed choline deficient diet; increase in TMAO levels in human subjects [189,190].
				Probiotic supplementation with *L. paracasei* F19	Re-establishment of microbiota diversity; protection against oxidative stress-induced liver damage in a rat model [170]
				Probiotic supplementation with *Lacticaseibacillus casei strain* Shirota	Increased abundance of *Bifidobacterium* and *Lactobacillus in* bacteria, alleviation of NAFLD symptoms (including altered expression of hepatic genes) in MCD diet-induced mice model [191].
Obesity/Metabolic syndrome	Decrease in fecal levels of *Bacteroides vulgatus; *increased abundance of *Actinobacteria*, *Firmicutes*, *Proteobacteria;* reduced abundance of *Bacteroides* and *Oscillospira* [187,192]	Increased [165]	Increased TMAO concentration in plasma and urine [165]	FMT	Microbiota re-modeling towards that of the donor, but no reduction in TMAO levels or improvement in metabolic markers [160].
				FMO3 enzyme inhibition	Reduced conversion of TMA into TMAO, improved lipid metabolism, and reduction in inflammation [181].
				Prebiotics = dietary fiber enriched diet	Reduced TMAO levels, microbiota re-modelling and improved metabolic markers in obese children [166].
				Arabinoxylan-oligosaccharide enriched prebiotic extract supplementation	Increased abundance of beneficial *Prevotella* bacteria and reduced choline availability for TMA synthesis in obese adults [165].
				Prebiotic supplementation with soluble dietary fiber	Reduction in TMA and TMAO metabolism (by 40.6%), increased abundance of beneficial bacteria, decreased weight gain, improved lipid and cholesterol markers in mice fed with red meat [164].

Abbreviations: CVD, cardiovascular disease; NAFLD, non-alcoholic fatty liver disease; SCFAs, short chain fatty acids; FMT, fecal microbiota transplantation; DMB, 3,3-dimethyl-1-butanol; MCD, methionine-choline-deficient.
